# Order out of Chaos: Elucidating the Opposing Effects of Air Pollutants and Heat on Blood Pressure

**DOI:** 10.1289/ehp.120-a76a

**Published:** 2012-02-01

**Authors:** Bob Weinhold

**Affiliations:** Bob Weinhold, MA, has covered environmental health issues for numerous outlets since 1996. He is a member of the Society of Environmental Journalists.

A body of research suggests that people with diabetes may be especially vulnerable to adverse cardiovascular effects of air pollution and heat, although the combined effects of these factors are not clear. Now an international team of researchers sheds light on potential impacts of this dual exposure [*EHP* 120(2):241–246; Hoffmann et al.]. In a study of the short-term effects of ambient air pollutants and temperature on arterial blood pressure in 70 people with type 2 diabetes mellitus, they found opposing effects—fine particulate matter (PM_2.5_) and black carbon in particular, were associated with increases in systolic blood pressure (SBP), whereas ozone and higher temperature were associated with decreases.

The study subjects, from the Boston, Massachusetts, area, ranged in age from 45 to 86 and were evaluated between September 2006 and August 2010. Each participant lived within 25 km of a central air pollution monitor. At each of 5 clinic visits spaced 2 weeks apart, participants answered questions and underwent blood and urinary analyses and clinical examinations. Mixed models that accounted for repeated measures in individual participants were used to estimate associations between air pollutants and temperature and blood pressure.

The researchers estimated that each interquartile increase in PM_^2.5^_ or black carbon in the 5 days prior to a clinical examination was linked with an average increase in SBP of 1.4 mmHg and 2.2 mmHg, respectively. In contrast, an interquartile increase in ozone was associated with a 5.2-mmHg decrease in SBP. Each increase of 11.5°C in mean temperature was associated with a 3.2-mmHg decrease in SBP before adjusting for ozone exposure. Estimated increases in blood pressure with PM_2.5_ exposure were greater in people with elevated baseline blood pressure, whereas associations with ozone exposure and temperature were greater in people with lower to normal blood pressure. Estimated differences in blood pressure tended to be progressively greater for each of up to 5 additional days of exposure prior to each clinical exam.

The researchers accounted for possible confounding factors such as sociodemographic variables, lifestyle indicators, prescription drug use, tobacco smoke, health history, and season. However, they were not able to estimate personal levels of exposure using the local and area monitoring stations, and factors such as physical activity were not addressed.

The authors note that the opposing effects may not cancel each other out and could plausibly act negatively in independent ways. Decreased SBP can be good in moderation, but it could be dangerous if lowered suddenly and significantly, especially in subjects with compromised vascular compensation mechanisms, which is common in diabetic patients.

